# Editorial: Insights in neuroinflammation and neuropathy

**DOI:** 10.3389/fnagi.2022.1060271

**Published:** 2022-11-10

**Authors:** Ching-Chi Chiu, Hsueh-Te Lee, Yu-Min Kuo

**Affiliations:** ^1^Department of Medical Biotechnology and Laboratory Science, College of Medicine, Chang Gung University, Taoyuan, Taiwan; ^2^Institute of Anatomy and Cell Biology, School of Medicine, National Yang Ming Chiao Tung University, Taipei, Taiwan; ^3^Department of Cell Biology and Anatomy, College of Medicine, National Cheng Kung University, Tainan, Taiwan

**Keywords:** oligodendrocyte, presbycusis, autoantibody, glaucoma, rapamycin, intracerebral hemorrhage

Neuroinflammation and neuropathy may result from the activation of immune cells in the nervous system and are a clinical features of various neurological and neurodegenerative disorders (Stephenson et al., 2018; Voet et al., 2019; Kwon and Koh, 2020). In addition to serving as a pathological marker, neuroinflammation is thought to play a crucial role in the development of neurpathologies, with activated microglia and astrocytes secreting inflammatory factors and neurotoxins that promote neuronal death (Stephenson et al., 2018; Voet et al., 2019; Habib et al., 2020; Kwon and Koh, 2020; Leng and Edison, 2021). To provide an overview of this topic, we have selected seven original research and review articles that delineate pathogenic processes and the role of neuroinflammation in a variety of neuropathies.

In one article, Li et al. review current research on and discuss the pathological presentation of myelin oligodendrocyte glycoprotein-IgG-associated disorder (MOGAD), a subtype of neuromyelitis optica spectrum disorder (NMOSD). MOGAD is an inflammatory disorder caused by antibody-mediated demyelination, and its clinical phenotypes overlap with other forms of NMOSD and multiple sclerosis. Importantly, patients with MOGAD have better response to immunotherapy and improved prognosis than those with non-MOGAD NMOSD. Additionally, there is no significant predominance of females in MOGAD. Brain MRI studies of MOGAD have revealed several neuropathological features, such as brainstem lesions, encephalopathy, and cortical encephalitis. This review provides new insights into clinical phenotypes of MOGAD.

In an original research report, Fuentes-Santamaría et al. demonstrate that age-related hearing loss and noise-accelerated presbycusis are closely related. They found that age and short/long-term noise interactions exacerbate presbycusis in young rodents by causing progressive dysfunction and promoting degeneration of ear cochlear cells and auditory structures. The degeneration occurs *via* common pathogenic mechanisms involving neuroinflammation and oxidative stress.

The presence of neuronal autoantibodies is associated with neuropsychological syndromes and cognitive impairment (Bartels et al., 2019, 2021). In a study conducted by Hansen et al., the application of methylprednisolone immunotherapy was assessed in patients with neural autoantibodies. Unfortunately, cognitive function and psychopathological symptoms in dementia patients with neural autoantibodies were not significantly improved by treatment with methylprednisolone for 6months. Larger cohorts or different experimental design may be needed to observe the potential benefits of immunotherapy in patients with neural autoantibody-associated cognitive impairment.

A review article by Watson et al. highlights and explores a scientific discrepancy between clinical intracerebral hemorrhage and most preclinical studies on the topic. While advanced age is a major risk factor for intracerebral hemorrhage, most research on the topic is conducted using young animals. Thus, additional studies should be performed with “old” animal subjects to dissect the mechanisms underlying aging-related susceptibility to intracerebral hemorrhage. Such an approach would greatly facilitate the development of novel treatment strategies and uncover knowledge gaps that currently limit clinical investigations.

Glaucomatous optic neuropathy is a feature of glaucoma that is characterized by progressive loss of retinal ganglion cells (RGCs) in the optic nerve. Degeneration of RGCs is known to be dependent on aging and elevated intraocular pressure, with structural changes in dendritic remodeling occurring in response to high intraocular pressure (Nakazawa and Fukuchi, 2020). To investigate how RGCs change in response to stimuli, the cells may be categorized according to their receptive field preferences for increments (ON-RGCs) or decrements (OFF-RGCs) of light. In their study, Lee et al. revealed that increases in intraocular pressure affected dendritic morphologies of RGCs in aging mice. Following the intraocular pressure-induced stress, OFF-RGCs in the eyes of young mice had better functional recovery than those in older mice. The delay in functional recovery of older eyes was attributed to loss of cellular adaptations in the RGCs. Thus, the study shows how aging influences function and morphology of RGCs.

Intracerebral hemorrhage is associated with high rates of morbidity and mortality (Li et al., 2021). Moreover, the recurrence rate in survivors is a major concern, as it affects 2% within 1 year and 9.6% within 5 years of the initial event. Compared to first-ever intracerebral hemorrhage, recurrent strokes are more fatal and disabling. Wan et al. followed a patient cohort for decades and assessed clinical and prognostic characteristics in patients experiencing recurrent and first-ever intracerebral hemorrhages. The authors found that recurrent stroke was significantly correlated with higher age and history of cardiovascular disease, including ischemic heart disease, ischemic stroke, hypertension, and hyperlipidemia. Moreover, recurrent intracerebral hemorrhage was an independent risk factor for 3-month poor outcome after adjusting for potentially confounding risk factors. Thus, even when controlling for risk factors such as blood pressure and blood lipids after the first intracerebral hemorrhage event, the risk of stroke recurrence is still higher than the risk of an initial event in a naive population.

To elucidate the neuroprotective effects of the immunomodulatory drug rapamycin in mitigating cisplatin-induced neurotoxicity and unpredictable cancer pain, Alotaibi et al. investigated whether rapamycin could prevent or significantly decrease cisplatin-induced neurotoxicity. The authors found that pretreatment with rapamycin increased levels of IL-17A and reversed CD8-T cell upregulation in mice treated with cisplatin. Additionally, rapamycin reduced the activity of caspase 3 in neurons, indicating a reduction in reduce neuronal apoptosis. This article suggests that rapamycin might be able to improve the quality of life for cancer patients.

We expect the selected articles will provide readers with an understanding of molecular mechanisms governing aging-related neuroinflammation and neuropathy, in addition to demonstrating how anti-neuroinflammatory strategies might help to limit pathogenesis of certain diseases.

## Author contributions

C-CC, H-TL, and Y-MK wrote the paper and commented on this manuscript. All authors contributed to the article and approved the submitted version.

## Funding

This work was supported by Chang Gung Memorial Hospital (CMRPD1M0291 to C-CC), National Science and Technology Council (109-2314-B-075-048-MY2 and 110-2320-B-A49A-508-MY3 to H-TL), and Yen Tjing Ling Medical Foundation (CI-109-12 and CI-110-20 to H-TL).

## Conflict of interest

The authors declare that the research was conducted in the absence of any commercial or financial relationships that could be construed as a potential conflict of interest.

## Publisher's note

All claims expressed in this article are solely those of the authors and do not necessarily represent those of their affiliated organizations, or those of the publisher, the editors and the reviewers. Any product that may be evaluated in this article, or claim that may be made by its manufacturer, is not guaranteed or endorsed by the publisher.
